# Concurrent chemoradiotherapy for squamous cell carcinoma of the anus using a shrinking field radiotherapy technique without a boost

**DOI:** 10.1038/sj.bjc.6600913

**Published:** 2003-04-29

**Authors:** A A Melcher, D Sebag-Montefiore

**Affiliations:** 1 Leeds Cancer Centre, Cookridge Hospital, Hospital Lane, Leeds, West Yorkshire LS16 6QB, UK

**Keywords:** anal cancer, chemoradiation, chemotherapy, radiotherapy

## Abstract

Chemoradiotherapy (CRT) is now widely accepted as the primary treatment modality for squamous cell cancer of the anus. While randomised trials have clearly shown CRT to be more effective than radiotherapy alone, there remains uncertainty over the optimal integration of chemotherapy and radiation. We describe a series of 50 patients treated by a site specialist gastrointestinal nonsurgical oncologist with CRT at a single UK centre. Chemotherapy comprised mitomycin C (MMC) (day 1) and 5-fluorouracil (5-FU) (days 1–4, and 29–32), concurrent with 50 Gy in 25 fractions radiation, using a two-phase shrinking field technique. A radiation boost was not planned. At a median follow-up of 48 months, 11 (22%) of the patients have failed locally, of which three have been surgically salvaged. Nine (18%) have died of anal cancer. These results are comparable with those from large randomised studies, and suggest that a two-phase shrinking field radiotherapy technique with no boost, concurrent with MMC/5-FU chemotherapy, is an effective regimen for this disease. The CRT regimen described here provides the basis for the ‘control arm’ of the current UK-randomised CRT trial in anal cancer (ACT2).

Chemoradiotherapy (CRT) is now established as the primary treatment modality for squamous anal cancer, with surgery reserved for persistent or recurrent disease. The pioneering work of Norman Nigro first showed in a case report that anal cancer was responsive to CRT (Nigro *et al*, 1974). The same institution subsequently published their experience of 45 patients treated with CRT using 30 Gy radiation combined with mitomycin C (MMC) and 5-fluorouracil (5-FU). Thirty-eight (84%) patients had evidence of histopathologically confirmed complete response on biopsy performed 6 weeks after completion of CRT (Leichman *et al*, 1985). Of these 38 complete responders, 89% remained disease free at a median follow-up of 50 months. Further evidence supported these results; Cummings *et al* reported their experience of 192 patients treated between 1958 and 1989 in seven small sequential studies. There were differences in radiotherapy fractionation, continuous *vs* split-course treatment and concurrent chemotherapy schedules (5-FU either alone or in combination with MMC). Although the numbers treated in each individual protocol were small, the combination of radiation, 5-FU and MMC appeared to give the highest rates of local control (Cummings *et al*, 1991).

Two large randomised trials, conducted by the United Kingdom Coordinating Committee for Cancer Research (UKCCCR) and the European Organization for Research and Treatment of Cancer (EORTC), demonstrated that CRT using concurrent 5-FU and MMC is superior to radiotherapy alone in the treatment of anal cancer, using the end points of local failure and colostomy-free survival (Anonymous, 1996; Bartelink *et al*, 1997). Furthermore, a Radiation Therapy and Oncology Group (RTOG) and Eastern Cooperative Oncology Group (ECOG) trial showed that MMC combined with 5-FU was superior to 5-FU alone when combined with radiation for colostomy-free survival (Flam *et al*, 1996).

These results from phase III randomised trials established the place of CRT in the treatment of anal cancer, and signalled the diminishing role of surgical resection in the primary management of this disease (Northover, 1991). Currently, the combination of 5-FU and MMC is accepted as standard chemotherapy within CRT for anal cancer, but the radiotherapy component of this combination treatment varies. The European trials employed an initial CRT treatment of 45 Gy, followed by a 6-week gap, and a boost with either external beam radiotherapy (15 Gy) or brachytherapy (25 Gy). In contrast, the RTOG trial reserved a boost only for those patients with biopsy-proven residual disease 6 weeks after initial CRT.

We chose to investigate a radiation regimen that treats macroscopic disease to 50 Gy and potential areas of microscopic disease to 30 Gy with a two-phase shrinking field technique using 2 Gy per fraction, and no boost. The rationale for omitting the boost is as follows. Firstly, there is no radiobiological basis for leaving a 6-week gap between initial CRT and delivery of a boost to the primary tumour. Indeed, such a gap during radiotherapy may be detrimental, allowing tumour cell repopulation. Secondly, the clinical evidence suggests that a boost may not be necessary. The RTOG trial showed that only 8% of patients had residual disease requiring a boost following biopsy confirmation 6 weeks after CRT (Flam *et al*, 1996), while in the UKCCCR trial, the minority of patients (approximately 10%) who did not receive their boost as part of planned treatment, had an outcome similar to those who did receive a boost (Anonymous, 1996). Although regional failure is potentially a significant problem in anal cancer, 30 Gy appeared to be adequate for even macroscopic disease in the series of 45 patients from Nigro's group, suggesting high intrinsic sensitivity of this disease to the CRT combination (Nigro *et al*, 1983). It is therefore reasonable to postulate that a dose of 30 Gy may also be adequate for microscopic disease. In addition, there is evidence that avoiding wide-field radiotherapy throughout CRT may significantly reduce long-term toxicity (Jenkins *et al*, 1995). Hence, we have chosen to use a two-phase shrinking field radiotherapy technique, restricting a higher dose (50 Gy) to macroscopic disease only. A simplified, prescriptive radiotherapy protocol without a boost, as described here, is also likely to lead to better compliance and improved quality assurance in future phase III trials.

## MATERIALS AND METHODS

Fifty patients with biopsy-proven squamous carcinoma of the anus were treated between March 1996 and December 1999. Patients were referred to a single clinical oncologist at the Leeds Cancer Centre, Cookridge Hospital, Leeds, from 20 surgeons in 11 surrounding hospitals. All patients were of World Health Organisation (WHO) performance status 0 or 1, and had been staged to exclude metastatic disease prior to treatment by chest X-ray and CT scan of abdomen and pelvis. None of the patients was HIV positive. Data are reported at a median follow-up of 48 months (range 29–73 months). Follow-up was performed jointly between the oncologist and the referring surgeon. Local failure was defined as biopsy-proven persistent or recurrent disease more than 3 months following definitive CRT. Pretreatment characteristics are given in Table 1

**Table 1:** Pretreatment characteristics

**Characteristic**	**Number**	**%**
*Age, years*		
<60	22	44
>60	28	56
Median	62	
Range	35–85	
		
*Sex*		
Male	18	36
Female	32	64
		
*Tumour stage*		
T1	2	4
T2	13	26
T3	31	62
T4	4	8
		
*Nodal stage*		
Node negative	37	76
Node positive	13	24
		
*Tumour site*		
Canal only	25	50
Canal>margin	12	24
Margin only	7	14
Margin>canal	6	12

. Tumour site was defined as exclusively anal canal or margin, or by the location of the majority of the disease (canal greater than margin, or margin greater than canal).

### Chemotherapy

Concurrent chemotherapy comprised MMC (8–12 mg m^−2^) on day 1 of radiotherapy, and 5-FU (750–1000 mg m^−2^) on days 1–4 and 29–32. Lower doses were used when clinically indicated according to individual patient age and frailty. All patients were considered fit for two-phase CRT, with creatinine clearance >60 ml min^−1^ (Cockroft/Gault estimate (Cockcroft and Gault, 1976) or ^51^CrEDTA (Chandler *et al*, 1969)), white cell count >3.0, neutrophils >1.2 and platelets >100. Fifteen patients received neoadjuvant chemotherapy (five patients one course, 10 patients two courses of cisplatin 80 mg m^−2^ day 1; 5-FU 800 mg m^−2^ days 1–4 q21 days) prior to definitive CRT, for symptomatic relief of severe local symptoms.

### Radiotherapy

Both phases of treatment were simulated at the same time. Patients were simulated and treated prone with a full bladder. All macroscopic primary tumour and involved nodes were considered for planning purposes as gross target volume (GTV). Node involvement was defined clinically and, for pelvic nodes, radiologically; fine-needle aspiration was not employed. The majority of patients were planned using orthogonal films throughout; small bowel opacification and rectal contrast were routinely used. However, a minority of patients required CT planning for phase II if they were unable to tolerate a rectal catheter for the insertion of rectal contrast. Macroscopic disease in the inguinal and femoral lymph node region or on the perianal skin was marked by wire. Moulded wax block bolus to the perianal skin was used for both phases of treatment.

**Figure 1:**
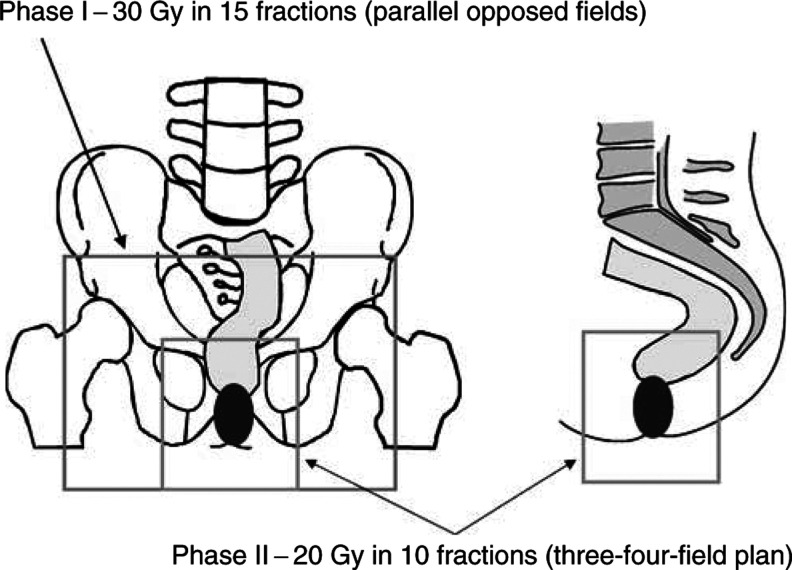
Radiotherapy treatment planning for a node-negative, anal canal tumour.

Phase I was the same for node-positive and -negative disease, and comprised large parallel opposed fields to include GTV and areas of potential microscopic disease including both inguinofemoral regions. The superior border was 0–2 cm above the bottom of the SI joints (=2 cm above the superior level of true bony pelvis). The lateral border was defined to cover both inguinal nodal regions, passing through the neck of the femora (Figure 1

). The inferior border was 3 cm below the inferior extent of the primary GTV, or the anal margin, whichever was more inferior. Phase I dose was 30 Gy in 15 fractions.

Phase II (node negative). Treatment was planned using orthogonal films. Treatment fields (three or four field plan) were designed to treat GTV with a 2–3 cm margin in all directions (Figure 1). For patients with disease confined to the anal margin, with no canal involvement, a single direct photon field was used to cover GTV with a 3 cm margin. The phase II dose was 20 Gy in 10 fractions.

Phase II (node positive). Parallel-opposed fields were used. Phase II of treatment was 20 Gy in 10 fractions, and covered GTV with a 3 cm margin in all directions.

Radiotherapy treatment planning (illustrated by fields for a node-negative anal canal tumour) is summarised in Figure 1.

In 11 patients, radiotherapy was given as a single phase using small fields throughout. In two cases this was because of early-stage disease (T1N0), and in nine patients because patients were assessed as too frail to tolerate skin toxicity from wide-field treatment. Although no boost radiotherapy was planned in this protocol, three patients did receive a further 15 Gy in six fractions of external beam radiotherapy 6 weeks after definitive CRT, because of the presence of persistent, palpable disease.

All patients were given prophylactic antibiotics (co-trimoxazole early in the series, then later ciprofloxacin) during CRT.

### Statistical methods

Local failure, disease-free survival and overall survival were estimated according to the Kaplan–Meier method (Kaplan and Meier, 1958).

## RESULTS

### Local and distant patterns of failure

In all, 47 patients completed CRT to the planned radiotherapy dose of 50 Gy. With a median follow-up of 48 months, the local failure rate for the whole group is 22% (11 patients). These local failures comprise 1 of 15 (7%) patients with T1/T2 disease, and 10 of 35 (28%) with T3/T4 disease. When local failure is considered with respect to nodal status, local failure has occurred in eight of 38 (21%) patients with node-negative disease, and three of 12 (25%) with node-positive disease. The three node-positive patients who relapsed had perianal, perianal and perirectal, and perineal disease with unilateral inguinal lymphadenopathy, respectively. All of the eight node-negative patients who relapsed locally did so with perianal recurrence without significant lymph node involvement.

Local failure occurred in four patients within 3 months of completion of CRT, four at 3–6 months, and three at greater than 12 months after completion of CRT. This suggests that the majority of patients failing locally do so early after treatment; the four patients who relapsed within 3 months probably never achieved remission.

**Figure 2:**
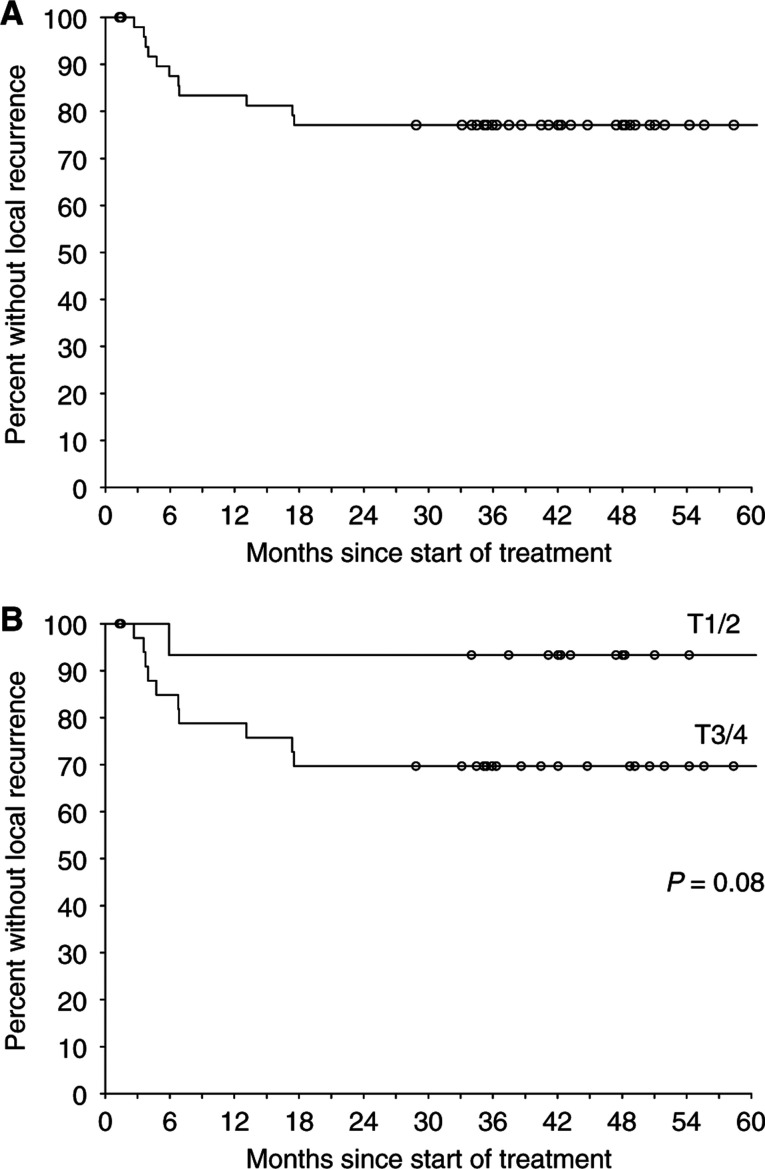
Actuarial freedom from local failure by (**A**) all patients and (**B**) T stage.

Eight patients have relapsed with distant metastases (of whom seven presented initially with T3/T4 disease) – of these, five have also failed locally. Sites of distant failure were liver (three patients), skin (one), perineum (one), bone (one) and mediastinal (one) or para-aortic lymph nodes (one). Local control and patterns of recurrence are summarised in Figure 2

and Table 2

**Table 2:** Patterns of failure

**Failure**	**Number**	**%**
Locoregional alone	6	12
Locoregional and distant metastases	5	10
Distant metastases alone	3	6
Deaths from anal cancer	9	18

.

### Colostomy

Nine patients have undergone anorectal excision for persistent or recurrent disease. Eight of these patients originally had T3/4 disease, and one had T2 disease. Three of these patients remain disease free to date, five have developed distant metastases and/or uncontrolled locoregional disease, and one has had two further local surgical excisions and is currently alive with locoregional disease. Three further colostomies have been performed, one due to bowel toxicity from treatment, and two in patients with poor anorectal function at presentation, which did not improve following CRT. The one patient with severe late radiation morbidity secondary to small bowel damage required colostomy and total parenteral nutrition. Radionecrosis has not been seen following treatment.

### Survival

**Figure 3:**
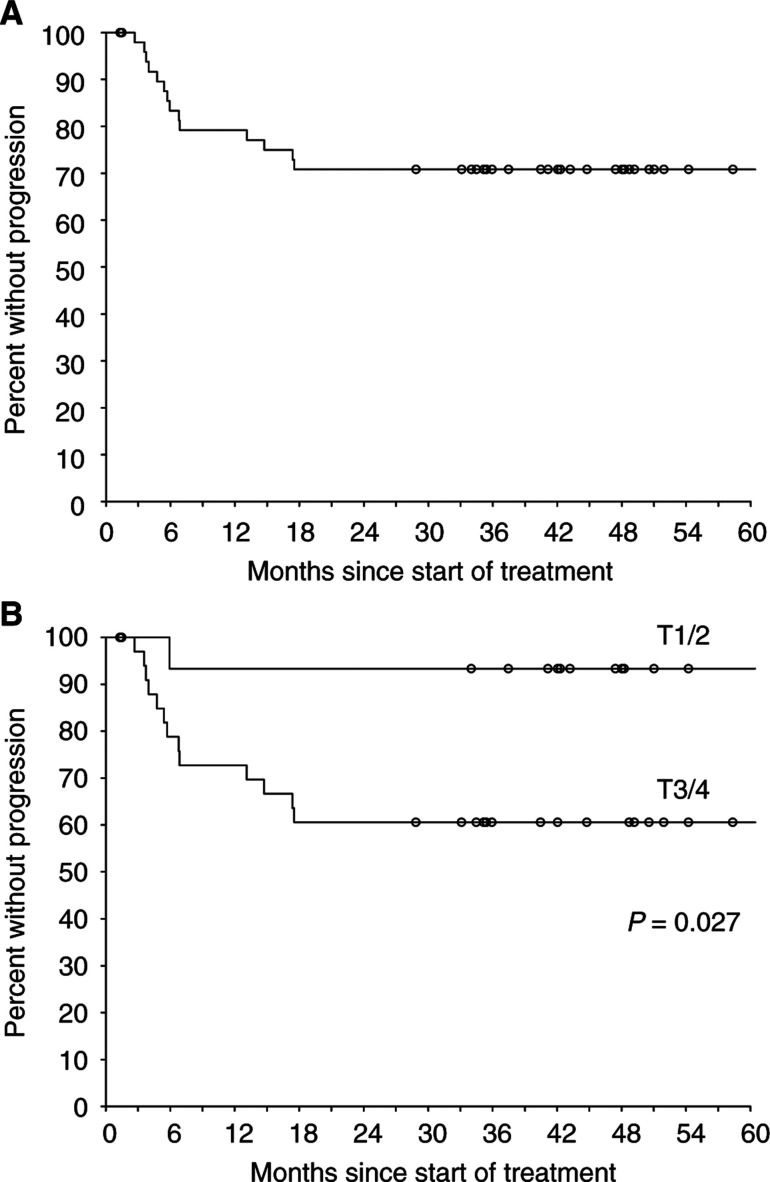
Actuarial disease-free survival by (**A**) all patients and (**B**) T stage.

Thirteen patients have died, nine of anal cancer. One patient who died postoperatively following a colostomy had stopped CRT at 30 Gy on the completion of phase I of treatment. One death occurred from apparently unrelated cardiac causes 20 days after the completion of CRT (development of an arrhythmia in a patient who had no cardiac symptoms previously or while on CRT), and a further patient who developed severe late bowel toxicity following treatment died 69 months after completion of CRT with no evidence of disease recurrence. The final death without recurrence was from an unrelated malignancy (carcinoma of the oral cavity) at 43 months from treatment. Data for disease-free and overall survival are shown in Figure 3

and Figure 4

**Figure 4:**
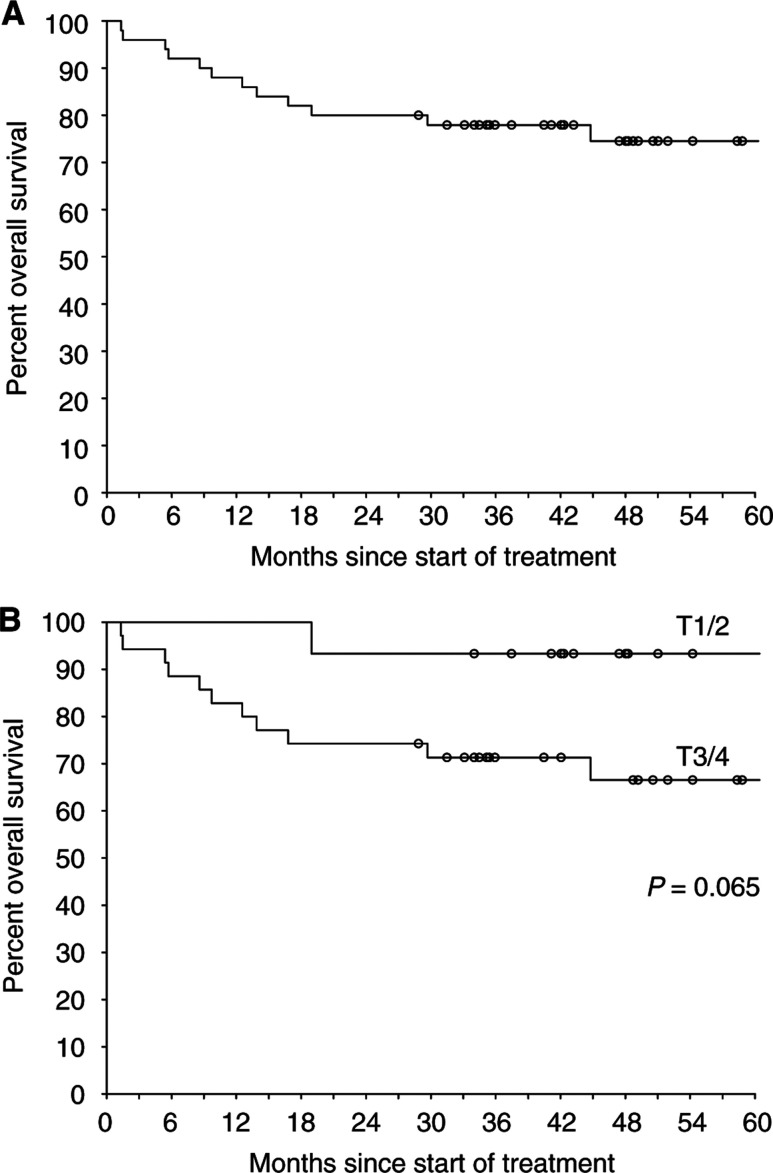
Actuarial overall survival by (**A**) all patients and (**B**) T stage.

.

### Radiotherapy compliance

Three patients received less than the planned radiotherapy dose of 50 Gy. In two patients, treatment was stopped slightly early (at 48 and 43.2 Gy) because of severe skin reaction, while in one elderly patient CRT was stopped after phase I (at 30 Gy) owing to general debility with treatment. Hence, 47 patients (94%) completed the full radiotherapy course. Only six patients (12%) had interruptions to treatment totalling four or more working days.

### Chemotherapy compliance

Modifications in the CRT chemotherapy regimen of MMC day 1 and 5-FU days 1–4 and 29–32 were as follows. Three patients received no MMC: one because of poor renal function, one because of development of chest pain (which was felt to be 5FU-related) during neo-adjuvant chemotherapy – this patient continued with cisplatin only in weeks 1 and 5 of CRT, and in one patient, the reason was not recorded. One patient received only 50% of the planned MMC dose, for unrecorded reasons. Regarding 5FU, the above-described patient with chest pain during induction chemotherapy received no concurrent 5-FU. All other patients received 5-FU at the planned dose during week 1 of CRT, but five patients were given no further 5-FU in week 5 (two because of low blood counts, one due to development of angina, one received cisplatin only in week 5 – reason unrecorded and in one patient all treatment was stopped after phase I). A further eight patients had their dose of 5-FU reduced in week 5 (two mucositis, two diarrhoea, two low blood counts, one severe desquamation and one unrecorded).

### Acute toxicity

The major acute toxicity was, as expected, severe moist skin desquamation. In two cases, radiotherapy was stopped early because of this, at 48 and 43.2 Gy. One other patient required a 13-day break in treatment because of skin toxicity, but completed the full radiotherapy dose. Acute skin toxicity was managed by Alevyn dressings and analgesia (usually opiates) as required. Otherwise, grade 3/4 toxicity comprised diarrhoea in seven patients (14%), neutropenia in four (8%) and thrombocytopenia in six (12%). There were no toxic deaths from treatment, or episodes of neutropaenic sepsis.

## DISCUSSION

Chemoradiotherapy is now widely accepted as the primary treatment modality for squamous anal cancer. Large randomised trials have now shown that CRT is superior to radiotherapy as a single treatment modality, and that the combination of MMC and 5-FU is effective as the chemotherapy component of CRT. However, several areas of controversy remain. The optimal radiation dose, fractionation and target volumes are uncertain, as are the roles of brachytherapy and temporal gaps in treatment. The most effective chemotherapy regimens to use during radiotherapy, and in the adjuvant setting also remain undecided. The incidence and severity of long-term toxicity of CRT is also becoming clearer as more mature series are reported (Myerson *et al*, 2001).

Nigro's original series used a dose of only 30 Gy, delivered using parallel-opposed fields at 2 Gy per day to cover the primary lesion with margins including the true pelvis and inguinal lymphatics (Nigro *et al*, 1983). The UKCCCR trial used 45 Gy over 4 or 5 weeks, again using anterior and posterior opposed fields (Anonymous, 1996). A target volume including the anus and inguinal lymph nodes was recommended, although the lymph nodes could be excluded as a unit policy. Six weeks following treatment, a clinical assessment of response was made, with good responders (greater than or equal to 50% response) recommended for boost radiotherapy (20–25 Gy iridium 192 implant or a further 16 Gy in six fractions EBRT), and poor responders (less than 50% response) considered for salvage surgery. The EORTC trial also used 45 Gy in 1.8 Gy fractions, delivered by a three- or four-field technique, with wider parallel-opposed fields specified only for those with established inguinal lymph node metastases (Bartelink *et al*, 1997). A boost of 15 Gy (for complete responders), or 20 Gy (for partial responders), was given at 6 weeks using photons, electrons or an iridium 192 implant. This contrasts with the RTOG trial, which used a complex shrinking field technique to 45 Gy in 1.8 Gy fractions, with field sizes reduced at 30.6 and 36 Gy (Flam *et al*, 1996). If the primary tumour was still palpable after 45 Gy, a further 5.4 Gy was given in three fractions. Inguinal lymph node involvement modified the target volume, such that the anterior field was extended to include both inguinal regions in N1 disease, with a further anterior electron or photon boost to bring the nodal dose at 3 cm depth to 50.4 Gy. A boost of 9 Gy to the primary tumour was reserved only for the 8% of patients with biopsy-proved residual disease 6 weeks after initial CRT, so that the majority of patients in this study received 45–50.4 Gy. The decision regarding total radiotherapy dose was determined by the biopsy result in this trial. Importantly, the complexity of the radiotherapy guidelines in the study led to protocol deviations in over 10% of patients.

Hence, radiotherapy protocols within anal cancer trials are of variable complexity and design, with no standard approach accepted. The three large randomised trials in this disease used a variety of radiotherapy planning techniques and doses, incorporating different lymph node coverage, single or multiple phases of treatment, and boost radiotherapy delivered with or without histological confirmation of persistent disease. With such differing protocols, effective comparison between trials becomes problematic, and quality assurance of specific radiotherapy techniques difficult, particularly for the more complex planning specifications.

In this series, we used a shrinking field radiotherapy technique to 50 Gy with MMC/5-FU concurrent chemotherapy. Forty-seven patients (94%) completed the radiotherapy protocol as planned, with only one patient having to stop treatment significantly early, at 30 Gy because of problems with poor anorectal function present prior to the start of CRT. Four patients had reduction or omission of MMC, and 13 (26%) modification of 5FU in week 5 of CRT. This compliance is comparable with the UKCCCR trial, in which 91% of patients completed planned radiotherapy in the CRT arm, and 74% received both courses of chemotherapy as planned.

Grade 3/4 toxicity was acceptable in this study, and there were no cases of neutropaenic sepsis or toxic deaths. There was one death within 3 months of treatment, from causes unrelated to the anal cancer or its treatment. Severe, long-term morbidity has so far been confined to one case of bowel toxicity requiring colostomy and total parenteral nutrition; this patient died without evidence of recurrence of disease 69 months after completion of treatment. Both these patients did not complete the full course of CRT. The patient who died within 3 months stopped treatment at 30 Gy because of general frailty, while the case with long-term bowel toxicity was stopped at 48 Gy due to severe skin toxicity. Since only three patients did not receive the full radiotherapy dose of 50 Gy, it is not possible to draw conclusions about the completion of planned radiotherapy and its effect on outcome or toxicity. The third patient, whose treatment was stopped at 43.2 Gy as a result of skin toxicity, remains disease free 55 months from completion of treatment. Similarly, the number and variety of chemotherapy modifications in a series of this size provides no clear guidance on any correlation between completion of the chemotherapy component of treatment, and outcome. There have been no cases of radionecrosis, consistent with other reports of a low prevalence of this complication following CRT (Dzik-Jurasz *et al*, 2001). However, we have not prospectively collected detailed quality-of-life information or other potential side effects of treatment, such as sexual dysfunction, which can be significant (Allal *et al*, 1999).

The rates of local failure in this study (22%) compare favourably with those seen in the 5-FU/MMC CRT arms of the large randomised trials (39% UKCCCR; 34% EORTC; 16% RTOG). Median follow-up is presently 48 months, and since most relapses in anal cancer occur within the first 2 years, significant worsening of these results is not anticipated. Indeed, of the 11 local failures to date in this series, eight occurred within just 6 months of completing CRT. There have been no locoregional failures within the 30 Gy treatment volume without failure either locally in the 50 Gy volume and/or disseminated disease. This is consistent with the hypothesis that 30 Gy is adequate for microscopic disease, which is further supported by a recent series in which 30 Gy seemed to be an effective dose in patients with anal cancer treated by excisional biopsy followed by CRT (Hu *et al*, 1999). However, there is evidence that 30 Gy is inadequate for control of macroscopic disease (Myerson *et al*, 1995). In this retrospective series, the local control rate for T2/3 patients treated to 30 Gy was 63%, compared to 77% for a dose of 40–50 Gy.

Since tumours with more advanced T stage and/or node involvement carry a poor prognosis, the question arises whether selected patients should be treated with escalating doses of radiation, and how boost treatments should be delivered (external beam or brachytherapy). Recent evidence in oesophageal cancer suggests that increasing the radiotherapy component of CRT may carry significant morbidity without benefit (Minsky *et al*, 2002). Consequently, in oesophageal cancer the recommended radiation dose from this series remains at 50.4 Gy. There is little evidence to support the use of a boost in anal cancer, since less than 10% of patients have persistent disease after a dose of 45–50.4 Gy (Flam *et al*, 1996), and the efficacy of any boost remains unproven (Anonymous, 1996). Moreover, recent evidence suggests that increasing the duration of any gap in split-course CRT for anal cancer has a negative impact on locoregional control (Weber *et al*, 2001). Therefore, the use of continuous external beam radiotherapy with no breaks in treatment, as described here, is a rational protocol design of CRT for anal cancer.

It is however important to establish whether cisplatin is superior to MMC when combined with 5-FU and radiation, and whether there is any benefit from the addition of two cycles of cisplatin/5-FU maintenance chemotherapy after completion of CRT. These questions are being addressed in a 2 × 2 factorial design in the current UK ACT2 trial.

In summary, this series shows that a shrinking field radiation technique with no boost is an acceptable component of radical CRT for anal cancer. Toxicity, colostomy, local failure and survival rates are consistent with previous data. The use of a clearly defined radiotherapy protocol is likely to improve compliance and simplify quality assurance in future CRT trials, in anal and other cancers. The technique described here is providing the basis for the radiotherapy technique in the ACT II trial.
